# How responsible leadership shapes followers’ low-carbon behavior: A dual-mediation model

**DOI:** 10.3389/fpsyg.2022.1086504

**Published:** 2023-01-09

**Authors:** Yihua Zhang, Xiyao Liu, Xiaoyan Zhang

**Affiliations:** ^1^Graduate School of Education and Psychology, Pepperdine University, Los Angeles, CA, United States; ^2^School of Business, Qingdao University, Qingdao, China; ^3^Business School, Beijing Technology and Business University, Beijing, China

**Keywords:** responsible leadership, environmental consciousness, environmental apathy, low-carbon behavior, leader-member exchange, social cognitive theory

## Abstract

**Introduction:**

In recent years, environmental problems such as global warming, rising sea levels, and species extinction have provoked a widespread concern all over the world, and many countries and international organizations have called for a reduction in carbon emissions. Theoretically, although many scholars have explored how responsible leadership influences subordinates’ work-related outcomes, little studies have examined the association between responsible leadership and employees’ low-carbon behavior. Therefore, to address this literature gap, we here drawing upon social cognitive theory developed a dual-mediation model to investigate how responsible leadership impacts employees’ low-carbon behavior, and through which mechanisms this impact may occur.

**Methods:**

By conducting a questionnaire survey in a company in China, we collected the valid data from 411 samples. Then using SPSS 26.0 and Mplus 8.1, we tested our proposed theoretical model and hypotheses by analyzing these data.

**Results:**

The empirical results showed that responsible leadership was positively related to employees’ environmental consciousness, which can further increase their low-carbon behavior. At the same time, responsible leadership was negatively related to employees’ environmental apathy, which can reduce their low-carbon behavior. And employees’ environmental consciousness and environmental apathy played the mediating roles in the relationship between responsible leadership and employees’ low-carbon behavior. Furthermore, we found that leader-member exchange (LMX) magnified the direct effect of responsible leadership on employees’ environmental apathy and strengthened the indirect effect of responsible leadership on employees’ low-carbon behavior via environmental apathy, but the moderating effect of LMX on another path was not significant.

**Discussion:**

These findings suggest that despite encouraging leaders to show responsible behaviors, promoting employees’ environmental consciousness and reducing their environmental apathy may be useful ways to facilitating their low-carbon behavior and achieving a low-carbon society. Moreover, establishing a high-quality of exchange relationship with followers may magnify the effectiveness of responsible leadership on lowering followers’ environmental apathy.

## Introduction

Recently, the research topic of responsible leadership has been attracting increased scholarly attention in the field of organizational behavior (Waldman and Galvin, 2008; Doh and Quigley, 2014; Dong and Zhong, 2022). Responsible leadership is a social and moral phenomenon that occurs in social processes of interaction (Maak and Pless, 2006a; Pless et al., 2012), defined as a “values-based and through ethical principles driven relationship between leaders and stakeholders who are connected through a shared sense of meaning and purpose through which they raise one another to higher levels of motivation and commitment for achieving sustainable values creation and social change” (Pless, 2007, p: 438). It was conceptualized on the basis of the intersection of corporate social responsibility and leadership literature (Doh and Quigley, 2014; Waldman and Balven, 2014). As an emerging leadership style, the most important difference between responsible leadership and other classical leadership styles, such as servant leadership, transformation leadership, humble leadership, authentic leadership, or ethical leadership, is its core notion of responsibility. That is, responsible leadership aims at not only facilitating employees’ positive outcomes, but also making the organizations responsible (Haque et al., 2019a,b).

Indeed, existing studies have drawn the conclusion that responsible leadership could generate desirable outcomes in the organizations. Specifically, as a form of value-based leadership style, responsible leadership has been found to be effective in promoting employees’ work-related attitudes and behaviors. For example, empirical research has showed the positive associations between responsible leadership and employees’ higher organizational commitment (Haque et al., 2019a), lower intention to quit (Haque et al., 2019b; Yasin et al., 2021), less unethical pro-organizational behavior (Cheng et al., 2019), and greater work engagement (Dong and Zhong, 2022). Besides, scholars also verified the significant effects of responsible leadership on performance. For instance, Lin et al. (2020) suggested that responsible leadership could positively influence employees’ job performance through the mediators of work engagement and helping initiatives. Javed et al. (2021) then confirmed the positive relationship between responsible leadership and corporate social performance at a firm level. Although great progress has been achieved in this field, how responsible leadership impacts employees’ low-carbon behavior still remains unresolved.

We believe that it is necessary to explore the influence of responsible leadership on employees’ low-carbon behavior. That is because, on the one hand, coping with this issue may theoretically enrich our understanding of the consequence of responsible leadership for employees’ non-work behaviors, and provide some guidances for organizations fostering responsible leaders. On the other hand, examining the predictors of employees’ low-carbon behavior has important practical implications. Specifically, recently environmental problems such as global warming, rising sea levels, extreme weather, and air pollution have becoming more serious and frequent than before, pushing many organizations and countries take actions or set policies in order to achieve low-carbon, green, and sustainable development (Arora et al., 2018). The Chinese government has announced the plan that China will aim to reach peak carbon dioxide emissions by 2030, and strive to achieve carbon neutrality by 2060. And research has indicated that the carbon emission of individuals’ daily behaviors accounts for around 80% of the total amount of global carbon emission (Bin and Dowlatabadi, 2005; Xia et al., 2022). Therefore, under such a context, it is essential to improve individuals’ low-carbon awareness and increase low-carbon behavior.

In this study, to fill this literature gap, we develop a dual-mediation model to investigate whether, how, and under which conditions responsible leadership may influence employees’ low-carbon behavior. The conceptual model in this study is based on social cognitive theory, which illustrates the interaction effects between external environment factor, individuals’ cognition, and individuals’ behavior (Bandura, 1986). One of the central propositions in social cognitive theory is external environment factors could exert a direct effect on individuals’ subjective cognitions, which then could shape their behavioral outcomes (Bandura, 1986, 2008). We believe social cognitive theory is a suitable framework to support our conceptual model as it has been found that leadership style can be viewed as an important external environment factor, which significantly impacted their followers’ subjective perceptions and behaviors (Pan, 2021; Deng et al., 2022). Thus, consistent with previous studies, we argue that responsible leadership may also have profound implications for employees’ cognitions and behaviors. More specifically, responsible leadership pays close attention to “society, the environment, sustainable value creation and positive change” (Han et al., 2019a, p: 306) and aims to achieving the coordinated development between people, society, and nature (Pless et al., 2011). In addition, responsible leaders not only practice social responsibility actively, but also set an example for their followers to focus on environmental issues and engage in environmental behavior (Pless, 2007; Han et al., 2019b). Given that leaders’ behaviors can affect their subordinates through daily interactions, we speculate that supervised by responsible leadership, employees’ environmental consciousness will be enhanced, which in turn, induces their low-carbon behavior. Meanwhile, we also propose that responsible leadership negatively predicts employees’ environmental apathy, which may have a negative association with their low-carbon behavior.

**Figure 1:**
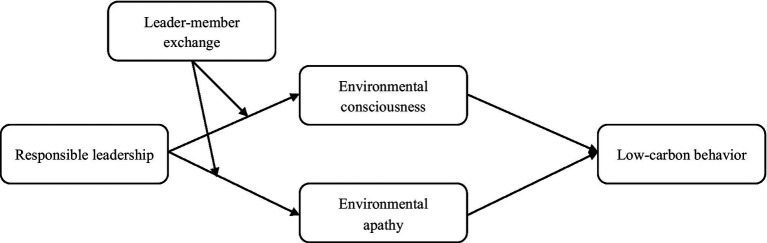
The theoretical model.

Besides, we go a step further to explore the boundary conditions that may alter the extent of responsible leadership influences employees’ cognitions and behaviors. According to social cognitive theory, employees’ individual difference may influence the process of environmental factors impacting individuals’ cognitions and behaviors (Wood and Bandura, 1989). Therefore, in this study, we propose that an individual characteristic in the organizational context, leader-member exchange (LMX), may play a moderating effect on the relationship between responsible leadership and employees’ outcomes. Followers’ perceived the quality of their exchange relationship with supervisors can be viewed as a salient individual feature that will affect the degree of their acceptance of responsible leaders as a role model, their identification of responsible leaders’ behaviors, and their willingness to follow responsible leaders. All these effects will be reflected on the employees’ responses to responsible leadership. In particular, we predict that employees with a high-quality of LMX relationship will be more likely to be affected by responsible leadership. That is, LMX positively moderates the relationships between responsible leadership and employees’ environmental consciousness and environmental apathy, and subsequently, moderates the indirect effect of responsible leadership on employees’ low-carbon behavior *via* environmental consciousness and environmental apathy. Figure 1 shows the theoretical model.

This study makes several theoretical contributions to current literature on responsible leadership and low-carbon behavior. Specifically, we firstly extend responsible leadership literature by shedding light on the effect of responsible leadership on molding employees’ low-carbon behavior. Although many previous studies have investigated the influences of responsible leadership on their subordinates’ work-related attitudes and behaviors, we know surprisingly little about how responsible leadership impacts their followers’ low-carbon behavior. By examining this association, it is also very helpful to deepen our knowledge of the antecedents of employees’ low-carbon behavior. In addition, based on social cognitive theory, we build a dual-mediation model to uncover the underlying mechanism through which responsible leadership may impact employees’ low-carbon behavior. In particular, we identify employees’ environmental consciousness and environmental apathy as the potential mediators that may link responsible leadership to employees’ low-carbon behavior. By doing so, we provide a new sight on understanding the consequences of responsible leadership for employees from the cognitive perspective. Moreover, we contribute to the current literature by exploring the boundary condition that may constrain the effect of responsible leadership on employees’ cognition and low-carbon behavior. Here, we examine the moderating effect of LMX on the direct relationships between responsible leadership and employees’ environmental consciousness and environmental apathy, as well as the indirect relationship between responsible leadership and employees’ low-carbon behavior *via* environmental consciousness and environmental apathy. Overall, we paint a more complete picture by clarifying under which conditions responsible leadership may maximally foster employees’ low-carbon behavior.

## Theory and hypotheses

### The mediating role of employees’ environmental consciousness

Responsible leadership is an emerging leadership style in which leaders build and sustain positive relationship with both of internal and external stakeholders to the organizations (Maak and Pless, 2006b). The stakeholders include employees, customers, communities, environment, suppliers, etc. (Pless, 2007). From the notion of responsible leadership, unlike other leadership approaches, responsible leadership aims at reaching the probable balance between achieving profit maximization and undertaking societal or environmental responsibilities (Miska et al., 2014), which reflects the inherent challenges and difficulties in responsible leadership. Environmental consciousness refers to the extent to which individuals’ beliefs value environmental problems (Ahmad et al., 2020). It reflects individuals’ attitudes toward their own behavior or others’ behavior with environmental consequences.

According to social cognitive theory, environmental factors can shape individuals’ subjective cognitions and further behaviors (Bandura, 1986, 2008). In the context of organizations, as followers and their leaders frequently interact with each other in daily work, their leaders’ behavioral styles may be an important external environmental factor in impacting followers’ perceptions and behaviors. Besides, existing studies have used social cognitive theory to explore the influences of leadership on employees’ outcomes. For example, Pan (2021) indicated the profound significance of paradoxical leadership in affecting followers’ paradoxical mindset and personal service orientation, and distal behavioral outcomes (i.e., OCB; Pan, 2021). And in one recent study, Deng et al. (2022) showed that ethic leadership could positively increase followers’ moral elevation and then their peer monitoring behavior (Deng et al., 2022). Thus, consistent with existing research, we propose that employees’ environmental consciousness may be a cognitive mechanism in explaining the relationship between responsible leadership and employees’ low-carbon behavior. In this section, we argue that responsible leadership may be positively associated with employees’ environmental consciousness.

First, as we noted above, responsible leaders not only focus on the organizations’ customers, suppliers, and community, but also see the natural environment as an important stakeholder. In other words, responsible leadership seeks the harmony between people, society, and environment. This may increase employees’ attention for environmental issues, their obligation for environmental protection, and corporate social responsibilities, thus promoting their environmental consciousness. Second, responsible leadership delivers a strong signal that they value environmental problems and sets a role model for the employees for actively engaging in eco-friendly behaviors. From the perspective of social learning, employees can observe and imitate leaders’ behaviors, finally internalize leaders’ values and thus increase their green shared vision. Furthermore, existing research has provided a series of evidence for this proposition. For example, many scholars found that responsible leadership could facilitate employees’ pro-environmental behavior (Han et al., 2019a,b; Afsar et al., 2020; Abbas et al., 2021; Tuan, 2022) and voluntary workplace green behavior (Zhang et al., 2021). Accordingly, we propose:

*H1a*: Responsible leadership will be positively related to employees’ environmental consciousness.

According to social cognitive theory, individuals’ subjective cognitions can shape their external behaviors (Bandura, 1986, 2008). Based on this, we propose that employees’ environmental consciousness may mold their low-carbon behavior. Low-carbon behavior refers to the behaviors that are helpful for building a low-carbon society such as using energy-saving appliances or turning off appliances when they are not in use (Whitmarsh et al., 2011). Previous studies have found that low-carbon knowledge (Lin and Yang, 2022), carbon neutrality behavioral intention (Zhao et al., 2022), and low-carbon awareness (Xia et al., 2022) can promote individuals’ low-carbon behavior. In this study, we argue that employees’ environmental consciousness can foster their low-carbon behavior for the following reasons:

First, environmental consciousness refers to a tendency to mentally reflect on the environment and to psychological conditions that reflect environmental commitment attitudes (Robert and Bacon, 1997). Scholars have noted that individuals with higher levels of environmental consciousness are concerned about the natural environment and are prone to perceive that they are responsible for the environmental protection (Huang et al., 2014). So they are more likely to show low-carbon behavior that has less harmful influence on the environment. Second, in an investigation into the tourists’ visiting intentions toward eco-friendly destinations, Ahmad et al. (2020) mentioned that essentially speaking, the idea of environmental consciousness incorporates the explicit psychological factors that link to individuals’ inclination to perform eco-friendly behaviors. Therefore, environmental consciousness provides a motivation for individuals to conduct low-carbon behavior. Furthermore, existing literature is in consensus that individuals’ high levels of environmental consciousness have the direct effects on their environment-friendly behaviors. For example, Schlegelmilch et al. (1996) found that the individuals endowed with a higher level of environmental consciousness contributed to their green purchasing decisions. Huang et al. (2014) showed that environmental consciousness could positively predict green customer behavior. In sum, we hypothesize:

*H1b*: Employees’ environmental consciousness will positively predict their low-carbon behavior.

Based on social cognitive theory, organizational factors could influence individuals’ subjective cognitions and then their subsequent behaviors (Bandura, 1986, 2008). In this study, responsible leadership has a positive influence on employees’ environmental consciousness. That is because, responsible leadership not only focuses on pursuing financial interests, but also undertaking environmental responsibilities at the same time (Lu et al., 2022). Through observing leaders’ responsible behaviors, employees may be impacted by the values of responsible leadership, and therefore pay a great concern on environmental problems by showing a higher level of environmental consciousness (Han et al., 2019a,b). As a result, elevated environmental consciousness may increase employees’ environmental felt-responsibility and motivate employees to conduct more environmental protection behaviors such as pro-environmental behavior, green behavior, or low-carbon behavior (Afsar et al., 2020; Abbas et al., 2021; Zhang et al., 2021). Taken together, we suggest that responsible leaders’ behavior can shape employees’ low-carbon behavior by molding their subjective cognition (i.e., environmental consciousness). Therefore, we make the following hypothesis:

*H1c*: Employees’ environmental consciousness will mediate the relationship between responsible leadership and employees’ low-carbon behavior.

### The mediating role of employees’ environmental apathy

After illustrating the mediation effect of employees’ environmental consciousness in the relationship between responsible leadership and employees’ low-carbon behavior, in this section, we try to reveal another path that may link responsible leadership to employees’ low-carbon behavior. Based on social cognitive theory (Bandura, 1986, 2008), we propose, responsible leadership may significantly influence employees’ environmental apathy, and subsequently their low-carbon behavior. Environmental apathy can be viewed as an individuals’ subjective cognition, which refers to “a lack of interest in environmental issues, and a general belief that problems in this area have been exaggerated” (Thompson and Barton, 1994, p: 151). Previous research has indicated that individuals’ general apathy toward environmental issues may be influenced by individuals’ own competitive worldview and narcissism personality (Abraham and Pane, 2016), support for free-market ideology (Heath and Gifford, 2006), ecocentrism and anthropocentrism (Thompson and Barton, 1994; Karpiak and Baril, 2008). Here, we argue that the level of employees’ environmental apathy may be impacted by their immediate supervisors’ responsible leadership style.

Specifically, first, as we mentioned above, responsible leaders aspire to be the true planetary citizens (Pless, 2007). That is, they hold the beliefs that they not only have responsibilities for the organizations’ performance, but also should not duck the responsibilities for caring for the environment (Voegtlin et al., 2012). Therefore, in the organizations, responsible leaders may proactively take actions to save material resources based on recycling principle. For example, they may call for using double-sided printing instead of single-sided printing and for the consideration of saving money. Besides, they may show many conserving behaviors such as energy, water or electricity. All of these actions reflect that responsible leadership put great value on environmental protection, thus contributing to awareness raising on ecological environmental issues among employees (Maak and Pless, 2006b). Hence, we speculate that supervised by responsible leaders, employees’ environmental apathy may be lowered to some degree. Furthermore, through the daily interaction with employees, responsible leaders will share amount of information about environment issues and highlight the importance of environmental protection. By doing this, employees can enrich their understanding of current conditions of environmental pollution or environmental governance, thus increasing their concerns for the environment. Given that environmental apathy means that individuals do not consider the environmental issues are important (Tortosa-Edo et al., 2014), we suggest that influenced by responsible leadership, employees’ apathy toward the environment will be broken effectively. To sum up, we hypothesize:

*H2a*: Responsible leadership will be negatively related to employees’ environmental apathy.

In existing literature, scholars have noted that environmental apathy or indifference is as destructive as other anti-environmental attitudes (Abraham and Pane, 2016). Accordingly, drawing upon social cognitive theory, we propose that employees’ environmental apathy may be negatively related to employees’ environmentally friendly behaviors such as low-carbon behavior.

Firstly, individuals’ apathy toward environmental issues reflects their “carelessness toward the protection and maintenance of the environment” (Gheith, 2013, p: 65). In other words, those individuals with high levels of environmental apathy show less concerns for the environment (Karpiak and Baril, 2008). Therefore, given that their unwillingness to pay attention to environmental issues, they are less likely to invest their personal resource such as time and energy to protect environment, cope with environmental problems, as well as show more pro-environmental behaviors: low-carbon behavior. Secondly, as a result of the individuals’ apathy toward environment, they may lack the knowledge about environmental problems, may not make accurate assessment of environmental problems, and thus will not place a high value on environmental protection. Heath and Gifford (2006) indicated that individuals’ environmental apathy had a detrimental effect on their beliefs about global climate change. Therefore, such an indifference attitude toward environmental protection may not increase individuals’ focus on whether their daily behaviors meet the requirements of environmental protection. Accordingly, they will not be intended to show low-carbon behavior in their daily life. Furthermore, in existing literature, relevant research has provided sufficient evidence for this speculation. For example, Kaltenborn and Bjerke (2002) showed that environmental apathy negatively predicted a preference for farm environments. Casey and Scott (2006) used the sample from Australian and found that level of apathy was negatively correlated with levels of pro-ecological behaviors. And Coşkun et al. (2022) demonstrated that apathy could prevent consumers from considering its environmental characteristic when purchasing products. To sum up, we propose the following hypothesis:

*H2b*: Employees’ environmental apathy will negatively predict their low-carbon behavior.

According to the research on leadership and social cognitive theory, as an important organizational factor, leadership style could cause profound implications for employees’ subjective perceptions and behaviors (Deng et al., 2022). In this section, we speculate that responsible leadership may exert a significant effect on employees’ low-carbon behavior by reducing their environmental apathy. Specifically, under the supervision of responsible leaders, employees may increase their focus on environmental problems, improve their realization that human beings are the subject of responsibility for protecting ecosystems, and then reduce their environmental apathy (Han et al., 2019b; Afsar et al., 2020; Abbas et al., 2021). When employees’ environmental apathy was decreased, they may show great willingness to concern environmental problems and great initiative in displaying environmental protection behaviors (Dai and Chen, 2021). In other words, such internal cognition will push them to exhibit external behaviors that are consistent with their subjective cognition, thus showing more low-carbon behavior. Taken together, we argue that employees’ decreased environmental apathy may be a potential mediator that can link responsible leadership to employees’ low-carbon behavior. Therefore, we propose the following hypothesis:

*H2c*: Employees’ environmental apathy will mediate the relationship between responsible leadership and employees’ low-carbon behavior.

### The moderating role of leader-member exchange

Leader-member exchange (LMX) refers to the dyadic relationship between a pair of supervisor and follower (Bauer and Green, 1996; Zhang et al., 2020). The formation of a LMX relationship is on the basis of a series of interpersonal interactions and exchanges of work-related resources (Graen and Cashman, 1975; Graen and Scandura, 1987). However, during the different relationship-building processes, two parties may both invest different levels of resources, thus causing the LMX relationships among each pair of supervisor and employee may distinct (Green et al., 1996; Wayne et al., 1997). That is, employees build different quality of exchange relationship with their supervisors, and accordingly, supervisors will not treat their employees in the same way (Zhang et al., 2020). Previous studies have demonstrated that a high-quality of LMX may contribute to employees’ innovative behaviors (Basu and Green, 1997), organizational commitment (Lee, 2005), job performance (Breevaart et al., 2015; Martin et al., 2016), and reduced turnover intention (Gara Bach Ouerdian et al., 2021).

Social cognitive theory indicated that individuals’ characteristics may influence the process of external environment factor impacting individuals’ cognitions and behaviors (Wood and Bandura, 1989). Based on this rationale of social cognitive theory, we propose employees’ responses to leadership may depend on the nature of LMX, that is employees’ LMX will positively moderate the influences of responsible leadership on their environmental consciousness and environmental apathy. Specifically, first, based on the principle of reciprocity, high-quality LMX relationships are characterized by mutual trust, respect, and liking (Liden and Maslyn, 1998). If employees’ LMX are high, they are inclined to perceive that their leaders treat them beyond the requirements of the organizations, show greater identification with or commitment to leaders, and define themselves as the in-group members. To reciprocate leaders’ treatment, those employees will be willing to follow leaders’ suggestions and requests. Therefore, employees with high LMX are more likely to internalize the values of responsible leadership, manifesting increased environmental consciousness and decreased environmental apathy. Second, employees with high-quality of LMX may keep frequent communications with their supervisors (Dienesch and Liden, 1986). The communications are more likely to deepen the influences of responsible leadership on the employees whose LMX is high, rather than whose LMX is low. Therefore, those employees with high-quality LMX will show more higher environmental consciousness and lower environmental apathy. Furthermore, relevant studies on LMX supported this proposition. For example, Michel and Tews (2016) found that LMX could accentuate the effects of leaders’ relations-oriented and change-oriented behaviors on employees’ OCB. Besides, Niu et al. (2018) found that LMX positively moderated the relationship between authentic leadership and employees’ relational identification with their leader. Overall, we argue that LMX could exaggerate the effectiveness of responsible leadership on employees. Thus, we hypothesize the following:

*H3a*: The effect of responsible leadership on employees’ environmental consciousness will be stronger for employees reporting higher LMX.

*H3b*: The effect of responsible leadership on employees’ environmental apathy will be stronger for employees reporting higher LMX.

### Moderated mediation effects

Going a step further, we argue that employees’ LMX may moderate the indirect effects of responsible leadership on employees’ low-carbon behavior through environmental consciousness and environmental apathy, respectively. As we noted before, employees with high-quality LMX will be more inclined to identify with their supervisors, be more likely to view their supervisors as the role model, and be more willing to imitate their supervisors’ behaviors (Tse et al., 2012; Hu and Liden, 2013). Therefore, we believe that the possibility of employees being influenced by responsible leaders is much higher for those employees who report high levels of LMX than those who report lower levels of LMX. In the current study, specifically, for those employees with a high-quality of LMX relationship, the positive effect of responsible leadership on their environmental consciousness and the negative effect of responsible leadership on their environment apathy will be exaggerated as they may internalize the values of responsible leadership effectively, thus reflecting in increased their low-carbon behavior. Accordingly, the mediation effects of environmental consciousness and environment apathy in the relationship between responsible leadership and low-carbon behavior will be strengthened when employees rate a higher level of LMX.

In sum, integrating the preceding discussion regarding the above hypotheses, we contend that for employees with high LMX, the indirect influences of responsible leadership on employees’ low-carbon behavior *via* environmental consciousness and environmental apathy will be higher. In contrast, for employees with low LMX, the indirect influences of responsible leadership on employees’ low-carbon behavior *via* environmental consciousness and environmental apathy will be lower. Thus, we hypothesize:

*H4a*: The indirect effect of responsible leadership on employees’ low-carbon behavior *via* environmental consciousness will be moderated by employees’ LMX, such that this indirect effect is stronger when employees’ LMX is high, but weaker when employees’ LMX is low.

*H4b*: The indirect effect of responsible leadership on employees’ low-carbon behavior *via* environmental apathy will be moderated by employees’ LMX, such that this indirect effect is stronger when employees’ LMX is high, but weaker when employees’ LMX is low.

## Materials and methods

### Samples and procedure

The sample of this study was full-time employees who worked in a large-scale manufacturing company in northern China. Manufacturing industry is one of the most representative industries in China. This company manufactured household appliances. We selected this company because it consumed a large amount of material resources, electricity, and energy in its daily production. The majority of our participants were front-line workers who worked in the factories of this company, and the others were employees who worked in a variety of departments, including administration, technology, marketing, finance, and operations. Because of the COVID-19 pandemic, we conducted this survey online. Specifically, first, under the assistant of human resource management department, we obtained the list of participants who were voluntary to join this survey. Then, all participants received an email in which we introduced this questionnaire survey, including explaining the procedure and the purpose of this survey, highlighting the importance of rating their actual feeling, and assuring data confidentiality. Besides, we also invited them to scan a QR code to join our research WeChat group. At the beginning of each wave of the survey, we shared the link of questionnaire in the WeChat group to ask all participants to completing the questionnaire.

To reduce the potential common method bias, we separate our data collection into two waves. At time 1, we asked the participants to report their perception of supervisors’ responsible leadership and leader-member exchange. Besides, they also provided their demographic information. In this stage, we totally distributed 509 questionnaires and received 435 responses. One month later in time 2, the participants rated on their environmental consciousness, environmental apathy, and low-carbon behavior. In this stage, we distributed 435 questionnaires and finally received 417 responses. After removing the responses who took less than half the average time to complete the questionnaire, randomly selected one option, and selected wrong choice for attention check item, we got 411 valid data with a response rate of 80.75%. Among the valid samples, 54.30% were male, and 45.70% were female. The majority of participants were between 26 and 45 years old, accounting for 62.50%. In terms of education, 33.80% held an associate degree and 30.90% of the participants held a bachelor degree or above. The details are given in Table 1.

**Table 1:** Demographics analysis.

**Demographics**		**Frequency**	**Percentage**
Gender	Male	223	54.30%
	Female	188	45.70%
Age	18–25	69	16.80%
	26–35	118	28.70%
	36–45	139	33.80%
	46–55	68	16.50%
	over 56	17	4.10%
Education	Junior high school degree or below	48	11.70%
	High school	97	23.60%
	Associate degree	139	33.80%
	Bachelor degree	95	23.10%
	Master degree or above	32	7.80%

N = 411.

### Measures

Following the suggestion of Brislin (1980), we translated the scales from English version to Chinese version. All of the items we used in this study were assessed on a 7-point Likert scale ranging from 1 (strongly disagree) to 7 (strongly agree), unless mentioned otherwise.

### Responsible leadership

Responsible leadership is a form of leadership style, which requires leaders to be morally conscious toward the stakeholders inside and outside of the corporation, manifesting appropriate decision-making, trust building, sustainable development, and green action choices (Zhao and Zhou, 2019). A 5-item scale developed by Voegtlin (2011) was used to measure responsible leadership. The sample item is “My supervisor considers the consequences of decisions for the affected stakeholders,” which was assessed on a scale from 1 = not at all to 7 = always (Cronbach’s α = 0.870).

### Environmental consciousness

Environmental consciousness refers to the degree to which individuals are concerned about environmental problems and are willing to make an effort to solve them (García et al., 2018). The participants reported their environmental consciousness using a 10-item scale from Alsmadi (2007). The sample item is “I get annoyed when someone contaminates the environment.” (Cronbach’s α = 0.936).

### Environmental apathy

Environmental apathy means that individuals are carelessness toward the environmental protection, lack interest in environmental issues, and are inclined to consider the environmental problems have been exaggerated (Thompson and Barton, 1994; Gheith, 2013). The participants reported their environmental apathy using a 9-item scale from Thompson and Barton (1994). The sample item is “I do not care about environmental problems” (Cronbach’s α = 0.920).

### Leader-member exchange (LMX)

LMX is a dyadic relationship that is built between a pair of supervisor and follower through a series of resources investment, and the high-quality of LMX relationship is characterized by mutual trust, respect, and liking (Bauer and Green, 1996; Liden and Maslyn, 1998; Zhang et al., 2020). We used a 7-item scale (Graen and Uhlbien, 1995) to measure employees’ perceived LMX. The sample item is “I have enough confidence in my leader that I would defend and justify his/her decision if he/she were not present to do so” (Cronbach’s α = 0.918).

### Low-carbon behavior

Low-carbon behavior refers to the behaviors that could impact the utility of substances or energy positively, and those would be able to change the structure and dynamics of an ecosystem positively (Li et al., 2020). The participants rated their low-carbon behavior with a 9-item scale from Bai and Liu (2013). The sample item is “I do not use disposable chopsticks” (Cronbach’s α = 0.950).

### Control variables

Consistent with previous research (Xia et al., 2022), we controlled employees’ demographic variables, including age, gender, and education levels.

## Results

### Confirmatory factor analysis

To test the discriminant validity of the constructs that were used in this study, we conducted a confirmatory factor analysis by using Mplus 8.1. As shown in Table 2, the five-model that consists of responsible leadership, environmental consciousness, environmental apathy, low-carbon behavior, and LMX shows the better fit indexes than other models (χ^2^ = 787.607, df = 730, χ^2^/df = 1.079, CFI = 0.994, TLI = 0.994, RMSEA = 0.014, SRMR = 0.043).

**Table 2:** Confirmatory factor analysis.

**Model**	**χ** ^**2**^	**df**	**χ** ^**2**^ **/df**	**CFI**	**TLI**	**RMSEA**	**SRMR**
Five-factor model: RL, EC, EA, LB, LMX	787.607	730	1.079	0.994	0.994	0.014	0.043
Four-factor model: RL+ LB, EC, EA, LMX	1462.336	734	1.992	0.930	0.926	0.049	0.067
Three-factor model: RL+ LB + EC, EA, LMX	2924.083	737	3.968	0.790	0.777	0.085	0.096
Two-factor model: RL+ LB + EC + EA, LMX	4531.124	739	6.131	0.635	0.615	0.112	0.124
One-factor model: RL+ LB + EC + EA + LMX	6783.262	740	9.167	0.419	0.388	0.141	0.155

N = 411. RL = responsible leadership. EC = environmental consciousness. EA = environmental apathy. LB = low-carbon behavior. LMX = leader-member exchange. Same for the following tables.

### Descriptive statistics

Table 3 shows the mean, standard deviations, and correlations of the variables. As expected, responsible leadership was positively related to employees’ environmental consciousness (*r* = 0.436, *p* < 0.01), but negatively associated with employees’ environmental apathy (*r* = −0.234, *p* < 0.01). Employees’ environmental consciousness is positively associated with their low-carbon behavior (*r* = 0.396, *p* < 0.01), while employees’ environmental apathy is negatively associated with their low-carbon behavior (*r* = −0.283, *p* < 0.01).

**Table 3:** Means, standard deviations, and correlations.

	**Mean**	**SD**	**1**	**2**	**3**	**4**	**5**	**6**	**7**	**8**
1. Gender	1.46	0.50	-							
2. Age	2.63	1.07	−0.034	-						
3. Education	2.92	1.14	−0.055	0.003	-					
4. RL	3.35	0.73	−0.007	−0.067	−0.098*	**(0.870)**				
5. EC	3.25	0.70	−0.101*	−0.092	0.021	0.436**	**(0.936)**			
6. EA	2.66	0.69	0.028	0.012	−0.038	−0.234**	−0.352**	**(0.920)**		
7. LMX	3.19	1.00	0.005	−0.069	−0.096	0.262**	0.290**	−0.142**	**(0.918)**	
8. LB	3.28	0.68	0.000	−0.033	0.004	0.447**	0.396**	−0.283**	0.243**	**(0.950)**

N = 411. Internal consistent reliability (alpha) coefficients are shown along the diagonal in bold italics. Gender, 1 = male, 2 = female. Age, 1 = 18–25 years old, 2 = 26–35 years old, 3 = 36–45 years old, 4 = 46–55 years old, 5 = over 56 years old. Education level, 1 = junior high school degree or below, 2 = high school, 3 = associate degree, 4 = bachelor degree, 5 = master degree or above. ** *p* < 0.01, * *p* < 0.05. Same for the following tables.

### Hypotheses testing

**Figure 2:**
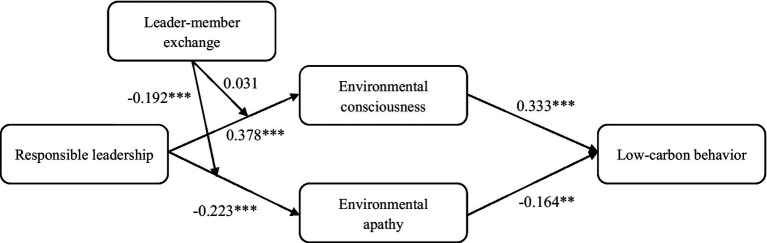
Unstandardized path coefficients from structural equation modeling results.

In this study, a structural equation model was conducted using maximum likelihood estimation along with 5,000 bootstrap estimations. The results in Table 4 have shown that responsible leadership has a positive impact on their subordinates’ environmental consciousness (*β* = 0.378, *p* < 0.001), and has a negative impact on their environmental apathy (*β* = −0.223, *p* < 0.001). Hence, Hypotheses 1a and 2a were supported. In addition, employees’ environmental consciousness positively influenced their reported low-carbon behavior (*β* = 0.333, *p* < 0.001), thus supporting Hypothesis 1b. Meanwhile, employees’ environmental apathy negatively influenced their own low-carbon behavior (*β* = −0.164, *p* < 0.01), supporting Hypothesis 2b. The results also indicated that employees’ environmental consciousness and environmental apathy both mediated the association between responsible leadership and employees’ low-carbon behavior (Table 4). For environmental consciousness, the indirect effect is 0.126 (95% CI = [0.078, 0.184]); for environmental apathy, the indirect effect is 0.036 (95% CI = [0.012, 0.072]). Therefore, Hypotheses 1c and 2c were supported. In addition, Figure 2 presents the final model with the empirical results.

**Table 4:** Regression results for directing and mediating effects.

**Predictor**	**Effect**	**S.E.**	**95% CI**	**Significance**
**M1: Environmental consciousness**				
X: Responsible leadership	0.378	0.046	[0.286, 0.469]	< 0.001
**M2: Environmental apathy**				
X: Responsible leadership	−0.223	0.046	[−0.312, −0.131]	< 0.001
**Y: Low-carbon behavior**				
M1: Environmental consciousness	0.333	0.050	[0.233, 0.427]	< 0.001
M2: Environmental apathy	−0.164	0.050	[−0.263, −0.066]	< 0.010
**Indirect effect of X on Y *via* M1**				
M1: Environmental consciousness	0.126	0.027	[0.078, 0.184]	< 0.001
**Indirect effect of X on Y *via* M2**				
M2: Environmental apathy	0.036	0.015	[0.012, 0.072]	< 0.050

N = 411. Effect = bootstrapped estimate. SE = standard error. LL = lower level. UL = upper level. CI = confidence interval. Same for the following tables.

**Figure 3:**
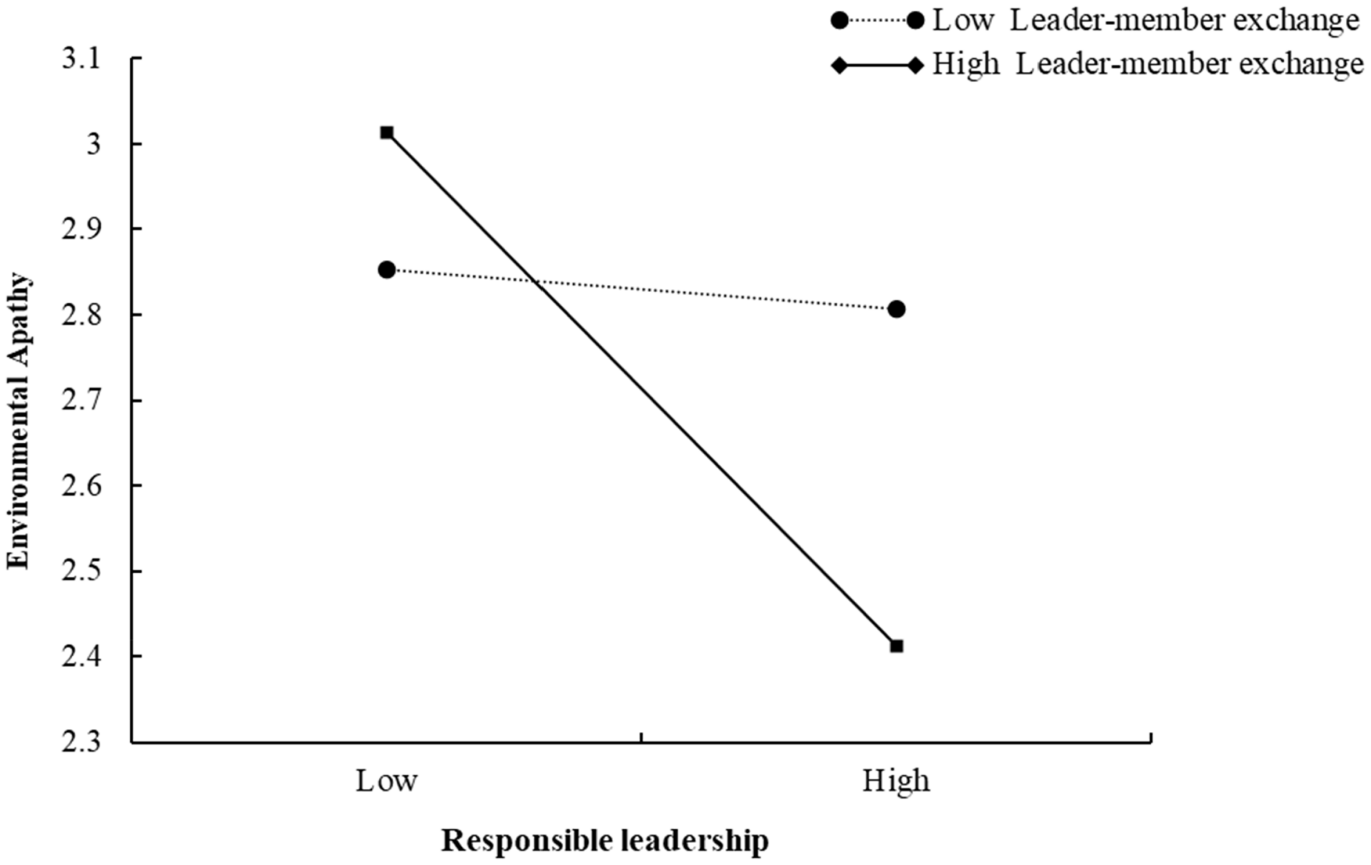
The moderating effect of LMX on the relationship between responsible leadership and employees’ environmental apathy.

Moreover, we tested the moderating role of leader-member exchange. As shown in Table 5, the interaction effect between responsible leadership and LMX is positively related to employees’ environmental apathy (*β* = −0.192, *p* < 0.001). But the influence of the interaction between responsible leadership and LMX on environmental consciousness is not significant (*β* = 0.031, *p* > 0.05). Figure 3 shows the simple slopes for different levels of LMX. In sum, Hypothesis 3b was supported, but Hypothesis 3a was not supported.

**Table 5:** Regression results for moderating effects.

**Predictor**	**Effect**	**S.E.**	**95% CI**	**Significance**
**M1: Environmental consciousness**				
X: Responsible leadership	0.378	0.046	[0.286, 0.469]	< 0.001
W: Leader-member exchange	0.135	0.031	[0.074, 0.194]	< 0.001
Interaction: X × W	0.031	0.042	[−0.054, 0.112]	n.s.
**M2: Environmental apathy**				
X: Responsible leadership	−0.223	0.046	[−0.312, −0.131]	< 0.001
W: Leader-member exchange	−0.058	0.034	[−0.125, 0.008]	n.s.
Interaction: X × W	−0.192	0.044	[−0.281, −0.105]	< 0.001

*N = 411*.

Furthermore, the empirical results in Table 6 confirmed that LMX moderated the indirect effect of responsible leadership on employees’ low-carbon behavior *via* their environmental apathy. Specifically, the mediation influence of environmental apathy in the relationship between responsible leadership and employees’ low-carbon behavior is stronger for employees who reported a higher level of LMX (i.e., conditional mediation effect = 0.068, 95% CI = [0.026, 0.123]) than those who reported a lower level of LMX (i.e., conditional mediation effect = 0.005, 95% CI = [−0.011, 0.032]), and the difference is also significant (difference = 0.063, 95% CI = [0.025, 0.115]). Hence, Hypothesis 4b was supported. However, although the mediation influence of environmental consciousness in the relationship between responsible leadership and employees’ low-carbon behavior is stronger for employees with high-quality LMX (i.e., conditional mediation effect = 0.136, 95% CI = [0.082, 0.202]) than those with low-quality LMX (i.e., conditional mediation effect = 0.116, 95% CI = [0.061, 0.179]), the difference is not significant (i.e., difference = 0.021, 95% CI = [−0.037, 0.074]). Therefore, Hypothesis 4a was not supported.

**Table 6:** Regression results for moderated mediating effects.

**Mediator**	**Leader-member Exchange**	**Effect**	**S.E.**	**95% CI**	**Significance**
M1: Environmental consciousness	−1 SD	0.116	0.030	[0.061, 0.179]	< 0.001
	Equal to Mean	0.126	0.027	[0.078, 0.184]	< 0.001
	+1 SD	0.136	0.031	[0.082, 0.202]	< 0.001
	Difference	0.021	0.028	[−0.037, 0.074]	n.s.
M2: Environmental apathy	−1 SD	0.005	0.010	[−0.011, 0.032]	n.s.
	Equal to Mean	0.036	0.015	[0.012, 0.072]	< 0.050
	+1 SD	0.068	0.025	[0.026, 0.123]	< 0.010
	Difference	0.063	0.023	[0.025, 0.115]	< 0.010

N = 411. Values for quantitative moderators are the plus/minus one SD from mean.

## Discussion

Based on social cognitive theory, this study built a dual-mediation model and revealed the underlying mechanisms through which responsible leadership may impact employees’ low-carbon behavior. The results demonstrated that responsible leadership was positively related to employees’ environmental consciousness, but negatively related to employees’ environmental apathy. Employees’ environmental consciousness significantly promoted their low-carbon behavior, while employees’ apathy was less likely to predict their low-carbon behavior. Besides, we also found the mediation effects of environmental consciousness and environmental apathy in the relationship between responsible leadership and low-carbon behavior. Furthermore, the results showed that LMX magnified the negative effect of responsible leadership on employees’ environmental apathy, and the indirect effect of responsible leadership on employees’ low-carbon behavior *via* environmental apathy. However, the moderating effects of LMX on the positive influence of responsible leadership on employees’ environmental consciousness and on the indirect influence of responsible leadership on employees’ low-carbon behavior *via* environmental consciousness were not supported. This result indicated that compared to employees’ environmental consciousness, employees’ environmental apathy may be more likely to be affected by the nature of LMX. That is, although employees with high LMX are inclined to follow the values of responsible leadership, they are more likely to show reduced environmental apathy, rather than increased environmental consciousness.

### Theoretical implications

Our study offers three theoretical contributions as follows. First, we contribute to the current literature by developing and testing a dual-mediation model to explore the influence of responsible leadership on employees’ low-carbon behavior. The majority of previous research has investigated the impacts of responsible leadership on employees’ work-related outcomes such as organizational commitment (Haque et al., 2019a), turnover intention (Haque et al., 2019b; Yasin et al., 2021), and job performance (Lin et al., 2020). However, as far as we know, to date, there is no study has focused on how responsible leadership affects employees’ low-carbon behavior. In addition, exploring the antecedents of individuals’ low-carbon behavior has important implications for building a low-carbon society and for achieving sustainable development. Therefore, we bridged this literature gap by building a conceptual model that could link responsible leadership to employees’ low-carbon behavior from the perspective of social cognitive theory. Our study responds to the call of Waldman and Balven (2014) by increasing the knowledge of the consequence of responsible leadership. Overall, we enrich the literature on responsible leadership by revealing whether, how, and under which conditions responsible leadership may shape employees’ non-work behaviors, enrich the literature on low-carbon behavior by shedding light on the important predicting role of responsible leadership, and finally expand the research scope of social cognitive theory into literature on responsible leadership and low-carbon behavior.

Second, this study revealed the underlying mechanism that could explain the influence of responsible leadership on employees’ low-carbon behavior by testing the mediating effects of environmental consciousness and environmental apathy. Existing literature has demonstrated that individuals’ environmental self-accountability (Xia et al., 2022), low-carbon knowledge (Lin and Yang, 2022), and carbon neutrality behavioral intention (Zhao et al., 2022) could positively predict their low-carbon behavior. However, relatively little research has explored whether responsible leadership may impact employees’ low-carbon behavior and how this impact occurs. To address this gap, in this study, we identified environmental consciousness and environmental apathy as two potential paths that may link responsible leadership to employees’ low-carbon behavior. The results showed that employees may internalize the values of responsible leadership by observing their effort in achieving sustainable development and green action choices (Zhao and Zhou, 2019). Influenced by leaders’ responsible behavior in the organizations, employees may increase their environmental felt-responsibility and their concerns about ecological environment, thus boosting their environmental consciousness and reducing their environmental apathy (Han et al., 2019b; Afsar et al., 2020; Abbas et al., 2021). Further, motivated by their increased value on environmental protection, they may show more low-carbon behavior in daily life (Casey and Scott, 2006; Huang et al., 2014). This study extends previous research by providing a reasonable explanation for why some employees show more low-carbon behavior and for how responsible leadership facilitate employees’ eco-friendly behavior through changing their perceptions about environmental issues.

Third, after illustrating the mechanism of responsible leadership impacting employees’ low-carbon behavior *via* environmental consciousness and environmental apathy, this study further answers the question of under which conditions responsible leadership may have the stronger or weaker effects on employees’ cognitions and behaviors. In particular, we examined the moderating effect of LMX on the relationship between responsible leadership and employees’ low-carbon behavior. The empirical results showed that compared with those employees with low LMX, employees with high LMX will tend to identify with their supervisors, be more likely to consider their supervisors as the role model, and be more willing to follow the environmental values of responsible leadership (Tse et al., 2012; Hu and Liden, 2013). Therefore, they may place a great value on environmental issues and show a lower level of environmental apathy than those employees who rated a low-quality of LMX. Furthermore, the mediation effect of environmental apathy in the relationship between responsible leadership and employees’ low-carbon behavior was strengthened. However, the results did not support the moderating effects of LMX on the path of responsible leadership on low-carbon behavior *via* environmental consciousness, which showed that the interact effect of responsible leadership and LMX could exert a more salient influence on reducing employees’ apathy than eliciting their consciousness toward the environment. Overall, in doing so, this study provides a more complete picture for understanding the effect of responsible leadership on employees’ low-carbon behavior.

### Practical implications

Our study not only investigated the influence of responsible leadership on employees’ low-carbon behavior through the dual-mediators of environmental consciousness and environmental apathy, but also provided some managerial suggestions for the organizations. First, this study found that responsible leadership could shape employees’ low-carbon behavior by increasing employees’ environmental consciousness and reducing their environmental apathy. This finding reinforced the necessary to focus on employees’ attitudes and cognitions toward the environmental issues. This is in line with previous studies, which have pointed that some individuals are less likely to show pro-environmental behavior as they hold the anti-environmental attitudes (Abraham and Pane, 2016). Therefore, in the organizations, to achieve the sustainable development and maintain the harmony between people, society, and environment, leaders should act as a responsible role model for their subordinates. Besides, leaders also could put more effort into improving employees’ environmental attitudes by sharing information about environmental problems, the urgency of environmental protection, and the significance of conducting low-carbon behavior.

Second, the finding that responsible leadership could significantly foster employees’ low-carbon behavior also provides some suggestions for the organizations. Specifically, in order to achieve sustainable development, build a green society, achieve peak carbon dioxide emissions by 2030, and reach the goal of carbon neutrality by 2060, organizations should pay close attention on fostering managers’ responsible leadership style. As leaders’ behaviors have a vital influence on employees’ behaviors and the organizations’ development. Therefore, managers at all hierarchies should be encouraged to learn the government’s requirements on the carbon emission, so as to reduce the organizations’ carbon emission in daily production and meet the requirements of environmental protection. Moreover, organization could design some training programs for managers to enhance their environmental awareness, establish their low-carbon values and increase their abilities to guide employees’ pro-environmental behaviors. Besides, the important characteristics that are embedded in responsible leadership could be used in selecting job hunters or promoting candidates.

Furthermore, this study indicated that high LMX can effectively magnify the negative effect of responsible leadership on employees’ environmental apathy, and further the mediation effect of environmental apathy in the relationship between responsible leadership and employees’ low-carbon behavior. This finding is consistent with previous studies, which showed that a higher level of LMX could exaggerate the effectiveness of leadership in improving employees’ attitudes and behaviors (Michel and Tews, 2016; Niu et al., 2018). Besides, it also emphasized the importance of building high-quality exchange relationships with employees. Hence, to foster employees’ low-carbon behavior, organizations could provide the opportunities for leaders and employees to increase their communications and cooperation such as arranging collective activities. And leaders should seek to build long-term working relationships with employees, endeavor to maintain the fairness when making decisions, and provide professional help or information for employees when they need, thus promoting employees’ trust and respect toward them. In doing so, a high-quality of LMX could maximize the effectiveness of responsible leadership on elicit employees’ eco-friendly behavior.

### Limitations and future research

Although this study has these above theoretical and practical implications, there still have some limitations. First, given that all the variables in our conceptual model were self-reported by employees, it may cause the concerns for common method bias. Thus, in order to test the detrimental influence of common method bias on our results, we conducted the Harman’s single-factor analysis and confirmed that the problem of common method bias is acceptable in this study. Even so, we encourage future research to exclude the potential impact of common method bias from many aspects. For example, future studies could use the supervisor-subordinate dyadic design that is measuring responsible leadership by using the data collected from supervisors or inviting other people who could observe employees’ daily behaviors (i.e., supervisor, coworker, or family) to rate employees’ low-carbon behavior.

Second, although we here examined the influence of responsible leadership on employees’ low-carbon behavior, the studies on the non-work outcomes of responsible leadership still remain infancy. Thus, we invite future research to investigate how the effects of responsible leadership spill over outside of the working domain. Besides, based on social cognitive theory, this study revealed the underlying mechanism thorough which responsible leadership could mold employees’ low-carbon behavior by identifying employees’ environmental consciousness and environmental apathy as two mediators. Scholars could conduct more research in the future to examine the mediating effects of other variables in the relationship between responsible leadership and employees’ low-carbon behavior. For example, future research could examine whether responsible leadership could promote employees’ low-carbon behavior by influencing employees’ CSR orientation or collectivist orientation.

Third, besides revealing the mediation mechanisms of responsible leadership impacting employees’ low-carbon behavior, we also examined the boundary conditions that could alter the degree of the above mechanisms. That is, we confirmed that employees’ perceived LMX not only positively moderated the direct effects of responsible leadership on employees’ environmental apathy, but also moderated the indirect effect of responsible leadership on employees’ low-carbon behavior *via* the above mediation. There have other boundary conditions that may influence the process of responsible leadership impacting employees. For example, future research could test the moderating effect of employees’ power distance orientation or employees’ leader identification on the relationship between responsible leadership and employees’ low-carbon behavior.

## Conclusion

Based on social cognitive theory, we developed a dual-mediation model to examine the influence of responsible leadership on employees’ low-carbon behavior. We found that responsible leadership could positively affect employees’ low-carbon behavior by enhancing employees’ environmental consciousness and reducing employees’ environmental apathy, respectively. Moreover, we also found the moderating effects of LMX on the direct relationships between responsible leadership and employees’ environmental apathy, and on the indirect relationship between responsible leadership and employees’ low-carbon behavior *via* environmental apathy.

## Data availability statement

The raw data supporting the conclusions of this article will be made available by the authors, without undue reservation.

## Author contributions

ZY and ZX contributed to the conception and design of the study. ZY organized the database. LX performed the statistical analysis. ZX wrote the first draft of the manuscript. ZY and LX wrote sections of the manuscript. All authors contributed to the article and approved the submitted version.

## Funding

This paper was supported by Zhang Mingyu Studio, Beijing Cultural Publicity High-level Talent Training Funding Project.

## Conflict of interest

The authors declare that the research was conducted in the absence of any commercial or financial relationships that could be construed as a potential conflict of interest.

## Publisher’s note

All claims expressed in this article are solely those of the authors and do not necessarily represent those of their affiliated organizations, or those of the publisher, the editors and the reviewers. Any product that may be evaluated in this article, or claim that may be made by its manufacturer, is not guaranteed or endorsed by the publisher.

## Supplementary material

The Supplementary material for this article can be found online at: https://www.frontiersin.org/articles/10.3389/fpsyg.2022.1086504/full#supplementary-material
