# Supplementary material for: How responsible leadership shapes followers’ low-carbon behavior: A dual-mediation model
Source: Front Psychol. 2023 Jan 9;13:1086504. doi: 10.3389/fpsyg.2022.1086504 (PMC9869243; doi:10.3389/fpsyg.2022.1086504)
Supplement: Supplementary file 1 [file Data_Sheet_1.docx]

**How responsible leadership shapes followers’ low-carbon behavior: A dual-mediation model**

**Appendix**

**Measurements**

**Responsible Leadership**

1. My supervisor demonstrates awareness of the relevant stakeholder claims.
2. My supervisor considers the consequences of decisions for the affected stakeholders.
3. My supervisor involves the affected stakeholders in the decision making process.
4. My supervisor weighs different stakeholder claims before making a decision.
5. My supervisor tries to achieve a consensus among the affected stakeholders.

**Environmental Consciousness**

1. I always advise others to keep the environment clean.
2. I get annoyed when someone contaminates the environment.
3. I respect all efforts to maintain and preserve the environment.
4. I appreciate living in a healthy and clean environment.
5. I respect rules and regulations to maintain and preserve the environment.
6. I always admire those who rationalize energy consumption.
7. I am aware of the impact of population explosion on the environment.
8. I realize that natural resources are scarce, thus must be used wisely.
9. I believe that man and nature have to be in harmony for survival.
10. I understand that the environment is for us and future generations, thus must be well maintained and preserved.

**Environmental Apathy**

1. Environmental threats such as deforestation and ozone depletion have been exaggerated.
2. It seems to me that most conservationists are pessimistic and somewhat paranoid.
3. I find it hard to get too concerned about environmental issues.
4. I do not think the problem of depletion of natural resources is as bad as many people make it out to be.
5. Most environmental problems willsolve themselves given enough time.
6. I do not care about environmental problems.
7. I’m opposed to programs to preserve wilderness, reduce pollution and conserve resources.
8. Too much emphasis has been placed on conservation.
9. I do not feel that humans are dependent on nature to survive.

**Low-carbon Behavior**

1. I save energy and resources.
2. I do not use disposable chopsticks.
3. I walk or ride bicycle for short trip.
4. I take public transportation for long trip.
5. I encourage family and friends to save energy and resource.
6. I participate in tree-planting campaign.
7. I encourage family and friends to participate in tree-planting campaign.
8. I support of low-carbon policies.
9. I discuss low-carbon issues with family and friends.

**Leader-member Exchange**

1. I know how satisfied my leader is with my work.
2. My leader understands my job problems and needs.
3. My leader recognizes my potential.
4. Regardless of how much formal authority my leader has built into his/her position, he/she would use his/her power to help me solve problems in my work.
5. Again, regardless of the amount of formal authority my leader has, he/she would “bail me out” at his/her expense.
6. I have enough confidence in my leader that I would defend and justify his/her decision if he/she were not present to do so.
7. My working relationship with my leader is effective.

**Table 1 Discriminate Validity (HTMT Criterion)**

| **Construct** | **RL** | **EC** | **EA** | **LB** | **LMX** |
| --- | --- | --- | --- | --- | --- |
| Responsible Leadership (RL) |  |  |  |  |  |
| Environmental Consciousness (EC) | 0.483 |  |  |  |  |
| Environmental Apathy (EA) | 0.260 | 0.378 |  |  |  |
| Low-carbon Behavior (LB) | 0.500 | 0.427 | 0.308 |  |  |
| Leader-member Exchange (LMX) | 0.288 | 0.308 | 0.151 | 0.259 |  |

*Note:* All findings satisfy the HTMT 0.85 criterion. HTMT = heterotrait-monotrait ratio.

**Table 2 Loadings and Cross Loadings**

| **Construct** | **Item** | **RL** | **EC** | **EA** | **LB** | **LMX** |
| --- | --- | --- | --- | --- | --- | --- |
| Responsible Leadership (RL) | RL1 | **0.757** | 0.214 | -0.144 | 0.222 | 0.083 |
|  | RL2 | **0.745** | 0.182 | -0.085 | 0.214 | 0.065 |
|  | RL3 | **0.726** | 0.204 | -0.047 | 0.166 | 0.107 |
|  | RL4 | **0.773** | 0.171 | -0.052 | 0.151 | 0.072 |
|  | RL5 | **0.757** | 0.184 | -0.046 | 0.243 | 0.172 |
| Environmental Consciousness (EC) | EC1 | 0.129 | **0.737** | -0.122 | 0.155 | 0.068 |
|  | EC2 | 0.132 | **0.780** | -0.161 | 0.121 | 0.032 |
|  | EC3 | 0.105 | **0.787** | -0.111 | 0.089 | 0.131 |
|  | EC4 | 0.045 | **0.783** | -0.100 | 0.185 | 0.109 |
|  | EC5 | 0.169 | **0.710** | -0.121 | 0.132 | 0.124 |
|  | EC6 | 0.128 | **0.754** | -0.152 | 0.153 | 0.117 |
|  | EC7 | 0.104 | **0.760** | -0.117 | 0.162 | 0.133 |
|  | EC8 | 0.141 | **0.740** | -0.090 | 0.144 | 0.125 |
|  | EC9 | 0.146 | **0.756** | -0.148 | 0.164 | 0.095 |
|  | EC10 | 0.098 | **0.767** | -0.100 | 0.092 | 0.060 |
| Environmental Apathy (EA) | EA1 | -0.018 | -0.125 | **0.779** | -0.081 | -0.058 |
|  | EA2 | -0.012 | -0.141 | **0.803** | -0.076 | -0.039 |
|  | EA3 | -0.024 | -0.142 | **0.766** | -0.046 | -0.026 |
|  | EA4 | -0.036 | -0.153 | **0.749** | -0.108 | -0.011 |
|  | EA5 | -0.032 | -0.084 | **0.791** | -0.101 | -0.051 |
|  | EA6 | -0.056 | -0.044 | **0.782** | -0.085 | 0.020 |
|  | EA7R | -0.165 | -0.304 | **0.559** | -0.243 | -0.150 |
|  | EA8 | -0.050 | -0.083 | **0.813** | -0.076 | -0.046 |
|  | EA9 | -0.108 | -0.128 | **0.790** | -0.077 | -0.016 |
| Low-carbon Behavior (LB) | LB1 | 0.103 | 0.177 | -0.056 | **0.774** | 0.096 |
|  | LB2 | 0.120 | 0.116 | -0.163 | **0.738** | 0.083 |
|  | LB3 | 0.098 | 0.117 | -0.094 | **0.768** | 0.029 |
|  | LB4 | 0.095 | 0.106 | -0.127 | **0.776** | 0.073 |
|  | LB5 | 0.128 | 0.111 | -0.074 | **0.750** | 0.161 |
|  | LB6 | 0.146 | 0.202 | -0.048 | **0.728** | 0.034 |
|  | LB7 | 0.100 | 0.105 | -0.090 | **0.745** | 0.112 |
|  | LB8 | 0.163 | 0.146 | -0.108 | **0.711** | 0.084 |
|  | LB9 | 0.151 | 0.192 | -0.064 | **0.706** | 0.024 |
| Leader-member Exchange (LMX) | LMX1 | 0.099 | 0.119 | -0.043 | 0.141 | **0.851** |
|  | LMX2 | 0.084 | 0.098 | -0.065 | 0.038 | **0.881** |
|  | LMX3 | 0.022 | 0.102 | -0.061 | 0.111 | **0.855** |
|  | LMX4 | 0.094 | 0.123 | -0.020 | 0.134 | **0.864** |
|  | LMX5 | 0.062 | 0.123 | -0.026 | 0.105 | **0.861** |
|  | LMX6 | 0.096 | 0.148 | -0.027 | 0.071 | **0.853** |
|  | LMX7 | 0.072 | 0.122 | -0.053 | 0.043 | **0.854** |
